# Molecular Mechanisms of Malignant Transformation by Low Dose Cadmium in Normal Human Bronchial Epithelial Cells

**DOI:** 10.1371/journal.pone.0155002

**Published:** 2016-05-17

**Authors:** Laura Cartularo, Thomas Kluz, Lisa Cohen, Steven S. Shen, Max Costa

**Affiliations:** 1 Department of Environmental Medicine, New York University School of Medicine, New York, New York, United States of America; 2 Department of Biochemistry and Molecular Pharmacology, New York University School of Medicine, New York, New York, United States of America; 3 Genome Technology Center, New York University School of Medicine, New York, New York, United States of Americ; University of Kentucky, UNITED STATES

## Abstract

Cadmium is a carcinogenic metal, the mechanisms of which are not fully understood. In this study, human bronchial epithelial cells were transformed with sub-toxic doses of cadmium (0.01, 0.05, and 0.1 μM) and transformed clones were characterized for gene expression changes using RNA-seq, as well as other molecular measurements. 440 genes were upregulated and 47 genes were downregulated in cadmium clones relative to control clones over 1.25-fold. Upregulated genes were associated mostly with gene ontology terms related to embryonic development, immune response, and cell movement, while downregulated genes were associated with RNA metabolism and regulation of transcription. Several embryonic genes were upregulated, including the transcription regulator SATB2. SATB2 is critical for normal skeletal development and has roles in gene expression regulation and chromatin remodeling. Small hairpin RNA knockdown of SATB2 significantly inhibited growth in soft agar, indicating its potential as a driver of metal-induced carcinogenesis. An increase in oxidative stress and autophagy was observed in cadmium clones. In addition, the DNA repair protein O^6^-methylguanine-DNA-methyltransferase was depleted by transformation with cadmium. MGMT loss caused significant decrease in cell viability after treatment with the alkylating agent temozolomide, demonstrating diminished capacity to repair such damage. Results reveal various mechanisms of cadmium-induced malignant transformation in BEAS-2B cells including upregulation of SATB2, downregulation of MGMT, and increased oxidative stress.

## Introduction

Cadmium is a toxic and carcinogenic transition metal nearly ubiquitous in the environment, as it naturally exists in the earth’s crust. It is also introduced into the environment via its many industrial uses. While a common route of human exposure is through diet, smokers and non-ferrous metal works are exposed to high levels of cadmium via inhalation. Each cigarette contains approximately 1.7 μg of cadmium [1] and human lungs can accumulate cadmium in concentrations of 0.9–6 μM [2]. Occupational exposure to cadmium has been linked to cancers of the lung, prostate, kidney, liver, hematopoietic system, bladder, pancreas, testis, and stomach [3], hence cadmium is classified as a class I human carcinogen by IARC [4,5]. However, the mechanisms of cadmium-induced carcinogenesis have yet to be fully defined. Cadmium is not mutagenic and does not form DNA adducts [6]. It is thought to induce oxidative stress by depleting glutathione and protein-bound sulfhydryl groups, leading to increased reactive oxygen species (ROS) production [7,8]. Cadmium may also act as an epimutagen via hypermethylation of gene promoters or by altering post-translational modifications to histones [3,9–12]

Special AT rich binding protein 2 (SATB2) is an embryonic transcription regulator with various roles in chromatin remodeling. SATB2 acts as a docking site for chromatin-remodeling enzymes such as histone acetylases and deacetylases and is involved in normal skeletal development. While SATB2 is typically only expressed in embryonic tissues, previous studies have found SATB2 to be upregulated in some human cancers and in BEAS-2B clones transformed by nickel, chromium (VI), arsenic, and vanadium [13]. The potential role of SATB2 in cadmium-induced malignant transformation has not been previously investigated.

Carcinogenesis is a multi-stage process that involves a multitude of alterations to the cell. DNA damage, if left unrepaired, can lead to gene mutations. These mutations may be detrimental if they occur in certain genes controlling cell cycle, proliferation, or other important cellular processes. Expression levels of various genes can also be altered via epigenetic mechanisms [14]. Many environmental and anthropogenic agents can cause DNA damage. Fortunately, our cells have evolved to deal with such toxic insults and many DNA repair pathways exist to ameliorate the damage induced by these agents. Alkylating agents are potent carcinogens that can be formed by burning tobacco or grilling certain foods. Alkylated DNA damage can also occur *in vivo* via certain enzymatic metabolites. The most carcinogenic of the alkylated DNA bases is O^6^-methylguanine (O^6^-meG) [15]. O^6^-meG is potentially mutagenic in that it typically pairs with adenine instead of cytosine, causing a G:C to A:T transition mutation. One pathway in which eukaryotic cells utilize to repair this lesion is via O^6^-methylguanine-DNA methyltransferase (MGMT). MGMT transfers the alkyl group from guanine onto a conserved cysteine in its active site. This renders the MGMT inactive and targets it for proteasomal degradation. MGMT promoter hypermethylation has been observed in the lymphocytes of humans exposed to high levels of polycyclic aromatic hydrocarbons [16], in the sputum of Chinese uranium miners exposed to high levels of radon [17], as well as in human bronchial epithelial cells transformed by nickel sulfide [18]. While cadmium ions have been found to inhibit transcription of the *E*. *coli ada* gene, which encodes O^6^-methylguanine-DNA methyltransferase [19], the effect of cadmium-induced transformation on MGMT expression has not been reported in human cells.

Smokers and metal workers are exposed via inhalation to high levels of cadmium [20], a known human lung carcinogen [21]. The goal of this study was to examine the carcinogenicity of low dose cadmium exposure on human bronchial epithelial (BEAS-2B) cells and to investigate the gene expression changes as well as other molecular events associated with cadmium exposure in order to better understand how this metal might cause transformation, even at fairly low doses.

## Materials and Methods

### Cell culture

Normal human bronchial epithelial (BEAS-2B) cells (ATCC, Manassas, VA) were cultured in DMEM, high glucose (Invitrogen, Carlsbad, CA), supplemented with 10% fetal bovine serum and 100 U/ml penicillin and 100 mg/ml streptomycin (Invitrogen). For cadmium exposures, cells were seeded one day prior to treatment. Cadmium chloride hemipentahydrate (Acros Organics, Gael, Belgium) was added to the media and evenly applied to the cultured cells. Cells were treated with 0.01, 0.05, or 0.1 μM cadmium. For chronic treatments, cadmium concentrations were maintained in the cell culture media. Cells were cultured in a 37°C incubator with 5% CO_2_ until harvesting. Cells were authenticated by Genetica DNA Laboratories (Burlington, NC) on July 22, 2015. Cells were matched 100% to 15 short tandem repeat (STR) loci and amelogenin to the reference profile of BEAS-2B (ATCC CRL-9609).

#### RNA extraction

At the end of the treatment, cells were washed twice with 1X PBS (Thermo Fisher Scientific, Waltham, MA) and collected in Trizol (Life Technologies, Carlsbad, CA). RNA was isolated and purified using RNeasy PlusMicro Kit (Qiagen, Valencia, CA) according to the manufacturers’ protocols.

### Real time PCR

500 ng of purified RNA was converted to single stranded cDNA using Superscript® III (Invitrogen). Quantitative real-time PCR analysis was performed using SYBR green (Applied Biosystems, Carlsbad, CA) on ABI prism 7900HT (Applied Biosystems). Relative gene expression levels were normalized to β-actin expression. All PCR reactions were performed in triplicate. Results were presented as fold change relative to the level expressed in untreated control cells using the ΔΔCT method [22]. Primer sequences used were as follows:

SATB2 forward: 5’-CAAGAGTGGCATTCAACCGCAC-3’

SATB2 reverse: 5’-ATCTCGCTCCACTTCTGGCAGA-3’

MGMT forward: 5’-GCTGAATGCCTATTTCCACCA-3’

MGMT reverse: 5’-CACAACCTTCAGCAGCTTCCA-3’

Beta actin forward: 5’-CACCATTGGCAATGAGCGGTTC-3’

Beta actin reverse: 5’-AGGTCTTTGCGGATGTCCACGT-3’

### Growth in soft agar

After six weeks of chronic exposure to cadmium, BEAS-2B cells were rinsed with PBS to remove the metal from the media then seeded in low gelling temperature Agarose Type VII (Sigma Aldrich, St. Louis, MO). 5x10^3^ cells were seeded in triplicate in 6-well plates in a top layer of 0.35% agarose onto a bottom layer of 0.5% agarose. Cells were allowed to grow for four weeks until individual colonies were large enough to select from the agar. Colonies were picked from each treatment and control group. These colonies were grown out into monolayers for four weeks. After monolayer growth, cells were collected in Trizol for RNA extraction for RNA sequencing (RNA-seq) or quantitative real-time PCR (qRT-PCR). A second set of plates was stained with INT/BCIP solution (Roche Diagnostics, Indianapolis, IN) for visualization and quantification of colonies, according to the manufacturer’s protocol.

### Scratch test migration assay

2.1x10^4^ cells were seeded in duplicate into each side of two-chambered culture inserts (Ibidi, Madison, WI) placed into tissue culture dishes and allowed to adhere for 24 hours until fully confluent. Inserts were removed using sterile forceps to create an even 500 μm cell-free gap. Cells were washed carefully with PBS to remove any floating cells and photographed 0, 6, and 18 hours post-gap using a Nikon HDMI-0a 1080P camera mounted onto a Nikon TMS-F phase contrast microscope.

### RNA sequencing

Cells were collected in Trizol (Life Technologies) and RNA was extracted according to the product insert. RNA was purified using Qiagen RNeasy Mini Kit. After fragmentation of mRNA, cDNA was synthesized and 3’ ends were adenylated. Ligated cDNA was then amplified using PCR and sent for processing. RNA-seq was performed by the NYU School of Medicine Genome Technology Center using the Illumina HiSeq2500 platform, 50 cycles with single-end reads. The alignment program, Bowtie (version 1.0.0) was used with reads mapped to the Ensemble GRCh37/hg19 (iGenome version) reference with two mismatches allowed. The uniquely-mapped reads were subjected to subsequent necessary processing, including removal of PCR duplicates, before transcripts were counted. Cadmium versus control differential gene expression was performed using the standard workflow from the DESeq2 R/Bioconductor package in the R statistical programming environment.

### Gene expression profiling

Top up- and downregulated genes were uploaded to The Database for Annotation, Visualization, and Integrated Discovery v 6.7 (DAVID; National Institute of Allergy and Infectious Diseases [23,24] and Ingenuity Pathway Analysis, version 1.4 (IPA; Qiagen) for pathway analysis.

### Whole cell lysis and protein isolation

Cells were lysed using boiling buffer (1% SDS, 1 mM Na_3_VO_4_, 10 mM Tris-Cl, pH 7.4). Briefly, 0.5 mL of 100°C preheated boiling buffer was added to each 10 cm cell culture dish at a sub-confluent density after two washes with 1X PBS. The lysate was denatured at 100°C for 5 minutes then sonicated using a Diagenode Bioruptor (Denville, NJ) at a maximum setting for 10 minutes. Samples were then centrifuged at 4°C for 15 minutes at 21,000 rpm. Protein was quantified using the Bio-Rad DC colorimetric Protein Assay (Hercules, CA) using bovine serum albumin (sigma) as a protein standard.

### Western Blot

Mini-PROTEAN TGX precast gels (Bio-Rad) were loaded with protein in Laemmli sample buffer (Bio-Rad) containing 5% (v/v) 2-mercaptoethanol (Sigma Aldrich). The Precision Plus Protein Kaleidoscope standard (Bio-Rad) was used to determine protein size. Electrophoresis took place in 1X tris/glycine/SDS buffer (Bio-Rad) at 100 volts at room temperature. The protein was transferred onto Immuno-Blot PVDF Membrane (Bio-Rad) in 1X tris/glycine buffer (Bio-Rad) at 22 volts overnight at 4°C. The membranes were blocked for 30–60 minutes with 5% (w/v) Blotting-Grade Blocker (Bio-Rad) in TBST at room temperature. Membranes were incubated with primary antibodies for one hour at room temperature or overnight at 4°C. Membranes were incubated with secondary antibodies (goat anti-mouse IgG-HRP [sc-2005, Santa Cruz Biotechnology, Dallas, TX] or goat anti- rabbit IgG-HRP [sc-2004, Santa Cruz Biotechnology]) for one hour at room temperature. Protein bands were detected using the Pierce ECL Western Blotting Substrate (Thermo Fisher Scientific). Relative band intensities were determined using ImageJ software (NIH).

### Stable SATB2 small hairpin RNA knockdown

Cadmium transformed-BEAS2-B clone 1 was cultured in DMEM supplemented with 10% FBS and 1% penicillin/streptomycin. Four SATB2 shRNA constructs (TG301833A, B, C and D) and scramble control shRNA plasmid (TR30013) were purchased from OriGene (Rockville, MD). Sequences of the four constructs were as follows:

shSATB2-A: 5’-TCCGCAATGCCTTAAAGGAACTGCTCAAA-3’;

shSATB2-B: 5’-GTTCAAAGTTGGAAGACTTGCCTGCGGAG-3’;

shSATB2-C: 5’-TGAACCAGAGCACATTAGCCAAAGAATGC-3’;

shSATB2-D: 5’-AATGTGTCAGCAACCAAGTGCCAGGAGTT-3’.

Plasmids were purified using a Qiagen QIAprep Spin Miniprep kit prior to transfection. Knockdown transfections were performed using Lipofectamine® LTX with PLUS reagent (Invitrogen) following the manufacturer’s protocol. Briefly, 150,000 cells were seeded into 6-well dishes 24 hours prior to transfection. The following day, 1 ug of purified plasmid was transfected into each well using 10 μL of Lipofectamine LTX and 2.5 μL of PLUS reagent per transfection. 24 hours post-transfection, the media was removed and replaced with fresh DMEM. After three days, 0.5 μg/ml of puromycin selection agent was added to the transfected cells. The cells were grown under selection for three weeks and harvested for western blot and real-time qPCR analysis to test for knockdown efficiency.

### Oxidative stress assays

Oxidative stress levels were determined by measuring the ratio of reduced glutathione (GSH)/ glutathione disulfide (GSSG), and protein carbonyl content. For the GSH/GSSG ratios, GSH/GSSG-Glo assay from Promega (Madison, WI) was used according to the manufacturer’s protocol. 10^4^ live cells were seeded in triplicate in white walled 96-well plates 24 hours before GSH/GSSG determination to allow the cells to attach. After 24 hours, cells were washed and lysed in the plate and GSH/GSSG ratios were measured by luminescence signal. Protein carbonyl content assay kit was purchased from Sigma Aldrich. Carbonyl content was determined by the derivatization of protein carbonyl groups with 2,4-dinitrophenylhydrazine leading to the formation of stable dinitrophenyl hydrazone adducts, which was then detected spectrophotometrically at 375 nm, proportional to the carbonyls present. The measurements were carried out in duplicate.

### Sodium butyrate and 5-aza-2'-deoxycytidine treatment

Control and cadmium clones were treated with either 10 μM 5-aza-2'-deoxycytidine for 48 hours, 5 mM sodium butyrate for 24 hours or with both inhibitors in DMEM. At the end of treatment, cells were collected in Trizol and RNA was extracted and converted to cDNA for qRT-PCR analysis.

### Temozolomide treatment

10^4^ cells were seeded in triplicate in 96-well dishes in 100 μL of DMEM. After 24 hours, cells were treated with 100 μM temozolomide (Sigma Aldrich) for 24, 48, or 72 hours. At the end of treatment, cell viability was measured using Roche Diagnostics Cell Proliferation Kit I, according to the product insert. Briefly, 10 μl of MTT labeling solution was added to each well and cells were incubated for four hours at 37°C to allow the formation of formazan salt crystals. After solubilization of the crystals, the plate was read at 575 and 690 nm on a SpectraMax plate reader (Molecular Devices, Sunnydale, CA) to determine cell viability.

## Results

### Chronic low dose cadmium treatment resulted in significant anchorage-independent growth in soft agar and cadmium-transformed clones exhibited increased migration ability

**Fig 1:**
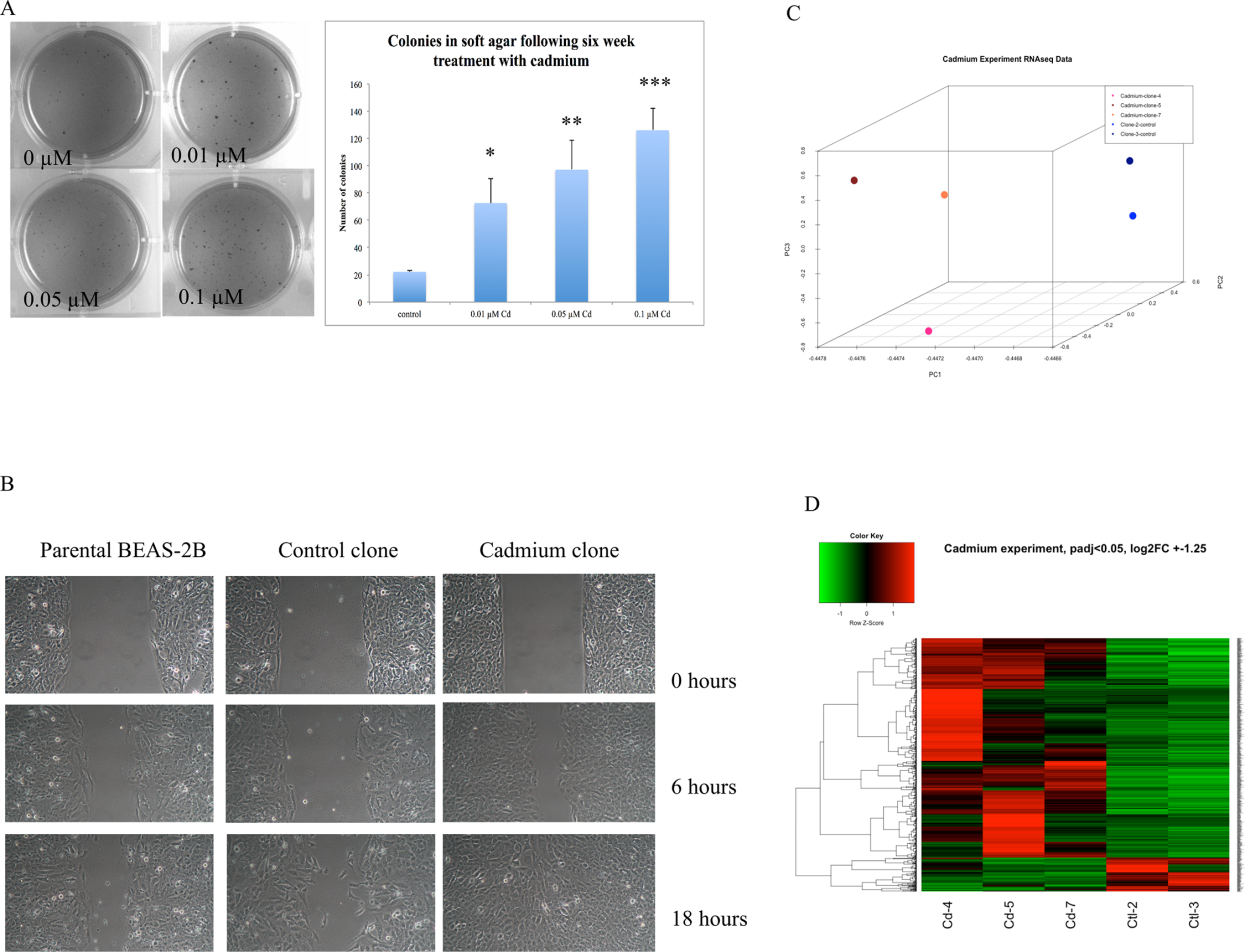
Significant dose-dependent anchorage-independent growth in soft agar was observed following six-week treatment with CdCl_2_. (A) Images shown represent two separate experiments that were seeded in triplicate wells and stained with INT/BCIP. Averages of experimental data are shown graphically (*p<0.005, **p<0.003, ***p< 0.0002). (B) Migration of cells in response to gap in monolayer. Cadmium clones exhibited increased migration ability compared to control clones and parental BEAS-2B cells. Scratch test images are representative of three control clones and 5 cadmium clones seeded in triplicate culture dishes. (C) Differential gene expression in cadmium clones versus control clones. Three-dimensional principle component analysis (PCA) of cadmium and control clones shows clear separation of cadmium-transformed (red) and spontaneous control clones (blue). (D) Hierarchical clustering of clones, all genes up- or downregulated 1.25-fold (padj>0.05), indicates the similarity between cadmium transformed clones and spontaneous clones. Red; upregulation, green; downregulation.

BEAS-2B cells treated for six weeks with varying sub-toxic doses of cadmium (0.01, 0.05, and 0.1 μM) exhibited significant dose-dependent anchorage-independent growth in soft agar (Fig 1A). Individual clones were picked from the agar and grown into monolayer cultures for all downstream experiments. Clones transformed by six-week treatment with 0.1 μM cadmium exhibited increased migration ability after a gap was generated in the monolayer compared to control clones and parental BEAS-2B cells, as assessed using the scratch test assay (1B). Results indicate that cadmium exposure, even at low doses, can lead to malignant transformation.

### Spontaneous control clones and cadmium-transformed clones exhibited differentially expressed genes

As shown on a three-dimensional plot of principal component analysis (PCA), cadmium-transformed clones and spontaneous clones showed clear separation and clustering according to sample type (Fig 1C). Control clones are shown in blue while cadmium-transformed clones are shown in red. These groups also separated via hierarchical clustering, shown for all genes changed over 1.25-fold (adjusted p<0.05) (Fig 1D). In total, 440 genes were upregulated over 1.25-fold, while 47 were genes were commonly downregulated in cadmium clones versus control clones. These top altered genes were then further analyzed using DAVID and IPA for gene expression profiling.

Top gene ontology (GO) terms associated with upregulated genes, as assessed by DAVID, included embryonic skeletal system development and morphogenesis, embryonic organ development and morphogenesis, skeletal system development and morphogenesis, locomotory behavior, integrin-mediated signaling pathway, lipid transport and localization, positive regulation by organism of innate immunity in other organism during symbiotic interaction, as well as other immune response terms, response to wounding, blood vessel development and morphogenesis, vasculature development, nucleosome and chromatin assembly, and inflammatory response (S1 Table). Top GO terms associated with downregulated genes included feeding behavior, regulation of RNA metabolism, regulation of transcription, DNA-dependent, classical pathway complement activation, and activation of plasma proteins involved in acute inflammatory response (S2 Table).

IPA revealed top networks associated with altered genes were cellular development, cellular growth and proliferation, hematological system development; metabolic disease, neurological disease, organismal injury and abnormalities; cancer, organismal injury and abnormalities, respiratory disease; protein synthesis, cancer; and skeletal and muscular system development and function, embryonic development, organismal development. The top molecular and cellular functions associated with the dysregulated genes included cellular movement, lipid metabolism, small molecule biochemistry, cellular development, and cellular growth and proliferation. Top upstream regulators were decitabine (5-aza-2'-deoxycytidine), amyloid beta A4 precursor protein (APP), transforming growth factor β (TGFβ), glucocorticoid receptor variant 1 (NR3C1), and mitogen activated protein kinase 1 (MAPK1).

### Special AT-rich binding protein 2 was upregulated in cadmium clones

**Fig 2:**
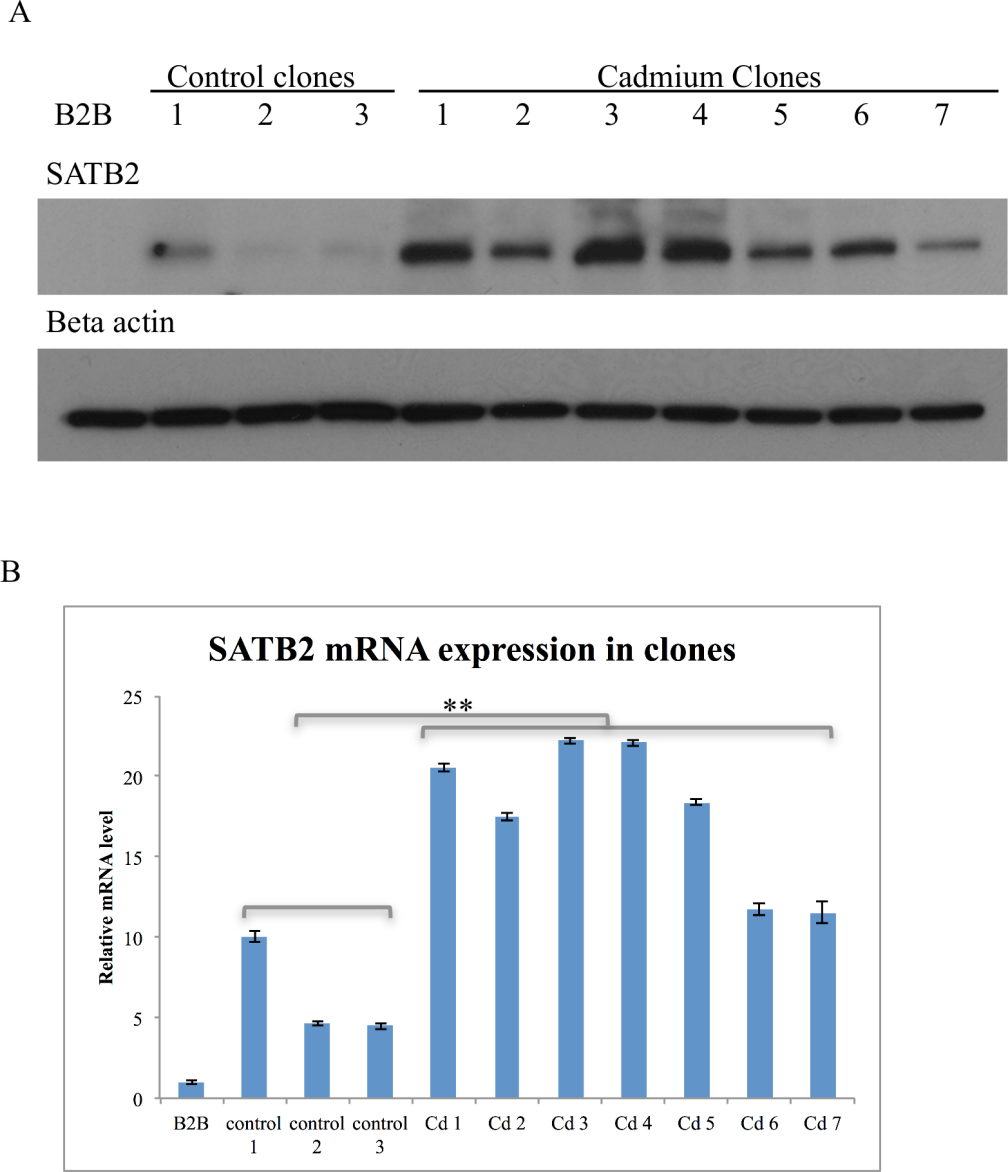
SATB2 overexpression in cadmium clones. SATB2 was overexpressed in all cadmium clones versus control clones and parental BEAS-2B (B2B) cells at the protein (A) and mRNA level (B). **p <0.001.

Special AT-rich binding protein 2 (SATB2), an important embryonic transcription regulator, was found to be upregulated in all cadmium clones versus control clones an average 3.6-fold according to RNA-seq results. Western blotting (Fig 2A) and qRT PCR (Fig 2B) further revealed that this gene was significantly upregulated in all cadmium clones that underwent soft agar selection as compared to control clones and parental BEAS-2B cells. SATB2 mRNA and protein were not induced in BEAS-2B cells after six weeks of treatment with 0.1 μM cadmium (data not shown).

### Cadmium transformed clones and BEAS-2B cells overexpressing SATB2 shared many commonly upregulated genes

BEAS-2B cells overexpressing SATB2 and cadmium-transformed clones shared 22% similarity (66/300) of top upregulated genes [25]. As determined by DAVID, the top GO terms associated with these commonly upregulated genes were cell adhesion, biological adhesion, negative regulation of transcription from RNA polymerase II promoter, positive regulation of epidermal growth factor receptor signaling pathway, neurotransmitter transport, response to inorganic substance, and negative regulation of transcription, DNA-dependent (S3 Table).

### SATB2 knockdown in cadmium clone 1 induced a clear phenotypic change

**Fig 3:**
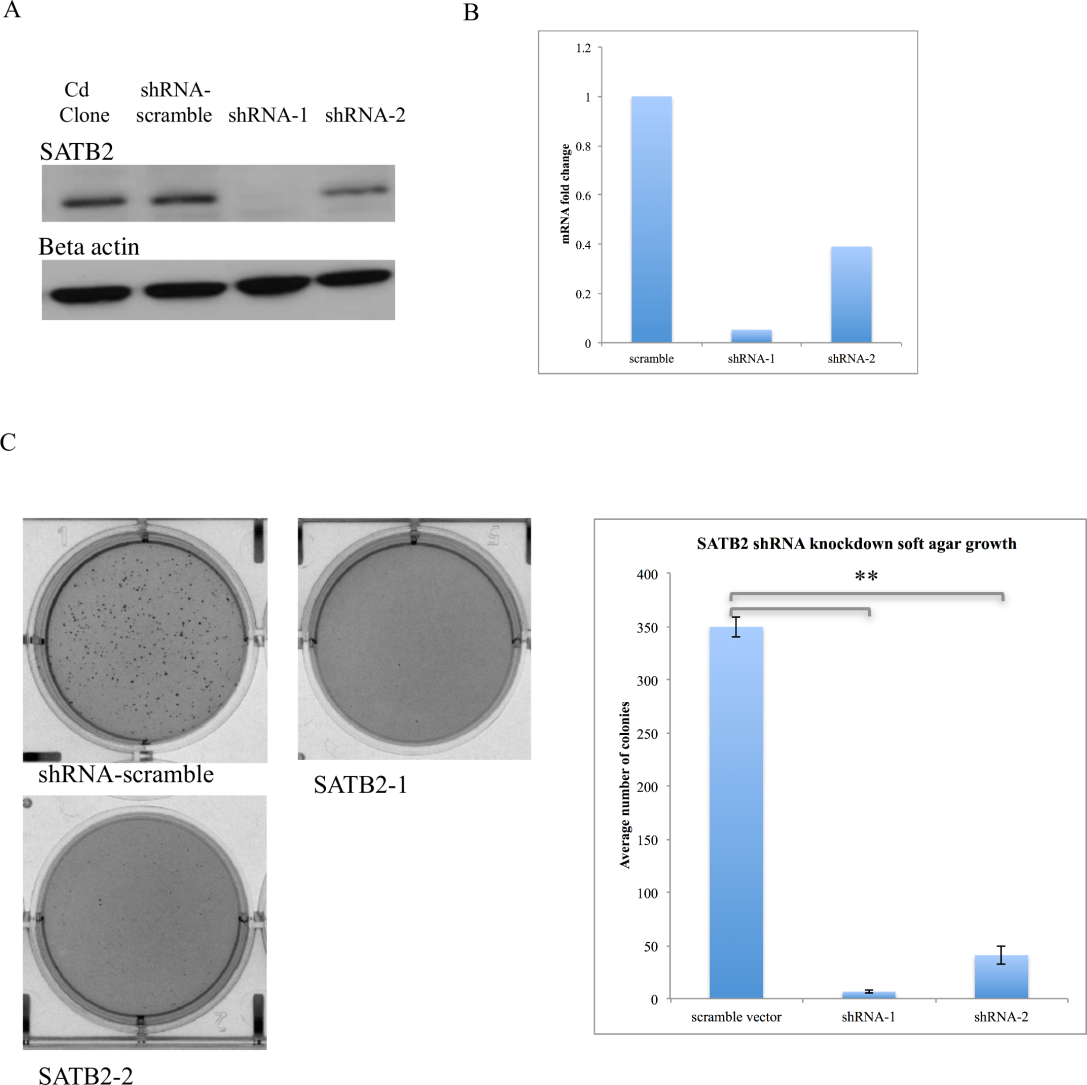
Stable SATB2 knockdown in cadmium clone 1 using small hairpin RNA. (A) SATB2 was effectively knocked down at the protein and (B) mRNA level using shRNA. Two individual knockdown clones are shown, with different levels of knockdown. (C) SATB2 shRNA knockdown significantly inhibited growth in soft agar. Both SATB2-shRNA clones significantly inhibited growth in soft agar. 5000 cells were seeded for each condition and figures are representative of cells seeded in triplicate wells. (** p< 0.0002).

Small hairpin RNA (shRNA) was used to stably knockdown SATB2 in a cadmium clone highly expressing SATB2 (cadmium clone 1). Fig 3A and 3B show that SATB2 was effectively knocked down at the protein and mRNA level. Two individual knockdown clones are shown (labeled as shRNA-1 and 2). These two clones exhibited varying levels of knockdown. shRNA-1 clone, which showed the greater level of knockdown (approximately 95% knockdown relative to scramble vector-transfected clone), exhibited a clear phenotypic change relative to unstransfected cadmium clone 1 and scramble vector-transfected cells (S1 Fig). These cells appeared rounder and more cuboid than the scramble vector cells. They also trypsinized much more easily and grew more slowly. shRNA-2 transfected cells did not exhibit this phenotypic change and this is likely due to the lower efficiency of SATB2 knockdown.

### SATB2 knockdown significantly inhibited growth in soft agar

SATB2 shRNA-transfected cells, as well as the scramble vector-transfected cells were seeded in soft agar to ascertain the effects of the knockdown on anchorage-independent growth. Both knockdown clones exhibited significantly reduced growth in soft agar relative to the scramble vector transfected cells. shRNA-1 transfected cells inhibited growth by nearly 98% (average of 6.67 colonies versus 350) (Fig 3C). The inhibition of soft agar growth displayed a dose-dependency with regard to the level of SATB2 expression.

### Cadmium transformed clones exhibited increased oxidative stress and autophagy markers

**Fig 4:**
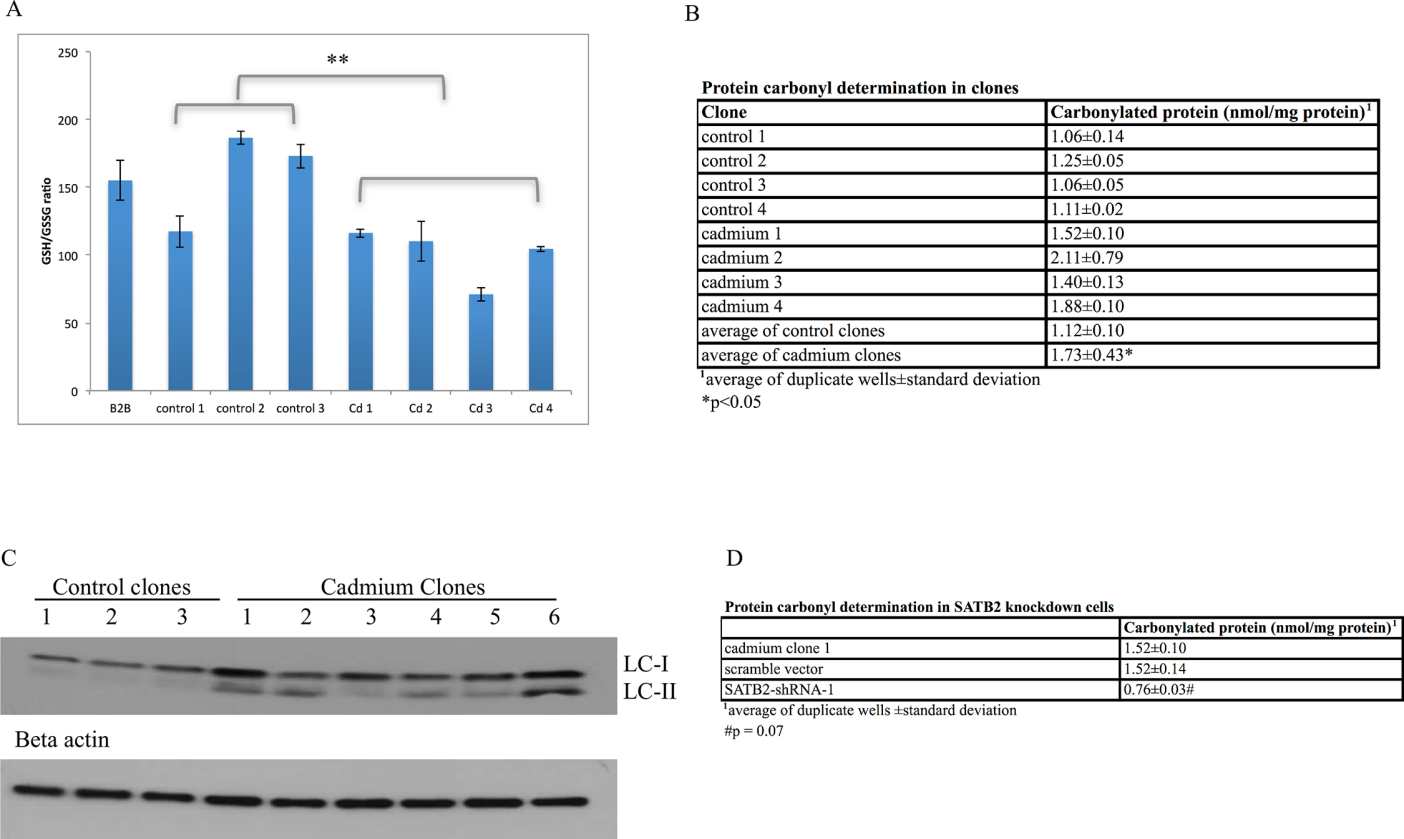
Increased oxidative stress was observed in cadmium clones versus control clones. (A) A very significant decrease in GSH/GSSG ratios was observed in cadmium clones versus control clones (**p< 0.001). (B) A significant increase in carbonylated protein was observed in cadmium clones relative to control clones (*p<0.05). (C) An increase in autophagy marker LC3A/B-II was observed in cadmium clones. During autophagy, LC3-I is converted to LC3-II via lipidation. (D) shRNA knockdown of SATB2 reduced the levels of carbonylated protein, however this was not significant (p = 0.07).

Oxidative stress was measured via determination of GSH/GSSG ratios as well as amount of carbonylated protein present. Cadmium clones exhibited a decrease in the ratio of reduced to oxidized glutathione (decrease in GSH/GSSG) (Fig 4A) as well as an increase in carbonylated protein levels (Fig 4B), both indicators of an increase in oxidative stress. The presence of autophagy marker LC3-II indicates increased autophagy in cadmium-transformed clones relative to control clones (Fig 4C). SATB2 shRNA knockdown reduced the amount of carbonylated protein present, however this reduction was not significant (p = 0.07) (Fig 4D).

### Cadmium clones exhibited reduced MGMT levels

**Fig 5:**
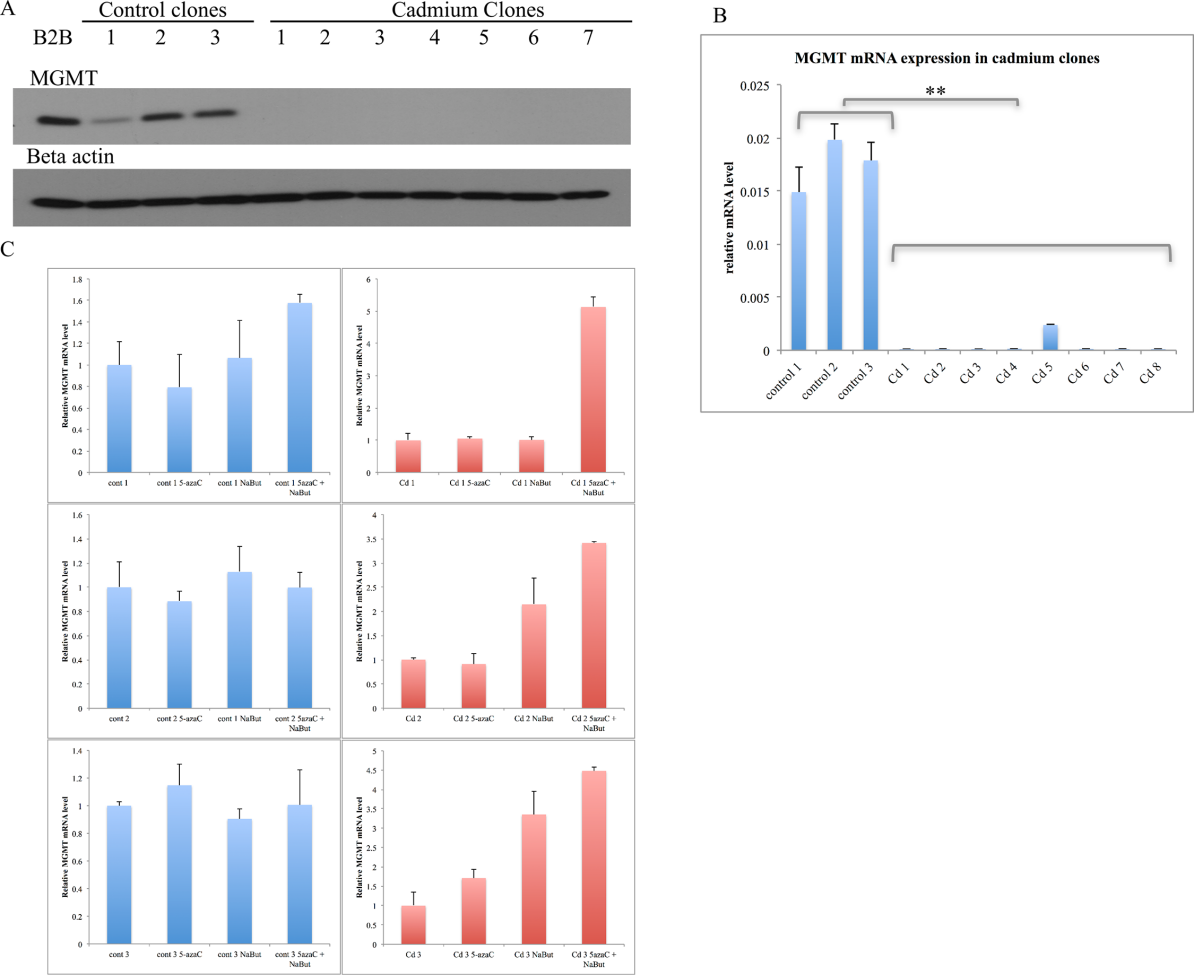
MGMT expression was depleted in all cadmium-transformed clones at the protein (A) and mRNA level (B) (**p < 0.001). (C) MGMT was epigenetically silenced in cadmium clones. Cells were treated with an inhibitor of histone acetylation (NaBut; sodium butyrate) or dna methylation (5azaC; 5-aza-2'-deoxycytidine), or a combination of the two, and MGMT mRNA levels were assessed by qRT-PCR. MGMT mRNA levels were upregulated in cadmium clones after treatment with the epigenetic inhibitors, but remained relatively unchanged in control clones.

RNA-seq data indicated that one of the most down-regulated genes in cadmium clones versus control clones was O^6^-Methylguanine-DNA Methyltransferase (MGMT). Western blotting and qRT-PCR revealed that this important DNA repair gene was depleted in all nearly cadmium clones versus control clones and parental BEAS-2B cells at the protein and mRNA levels (Fig 5A and 5B). MGMT levels decreased only slightly in BEAS-2B cells after six weeks of treatment with 0.1 μM cadmium (S2A and S2B Fig), indicating that this important change appeared to occur only in those cells that were selected for growth in soft agar after chronic treatment.

### MGMT was epigenetically silenced in cadmium clones

To ascertain whether MGMT was downregulated via epigenetic mechanisms in cadmium clones, cells were treated with inhibitors of DNA methylation (5-aza-2'-deoxycytidine) or deacetylation of histones (sodium butyrate), either separately or in combination, and MGMT mRNA levels were assayed for reactivation. qRT-PCR results indicated that treatment of cadmium clones with the combination of these inhibitors of epigenetic silencing was able to induce MGMT mRNA levels 3.5–5.1-fold (Fig 5C, left panel). Control clones treated with 5-aza-2'-deoxycytidine or sodium butyrate did not exhibit a significant change in MGMT mRNA levels (Fig 5C, right panel).

### Cadmium clones are less efficient at repair of alkylated DNA damage induced by temozolomide

**Fig 6:**
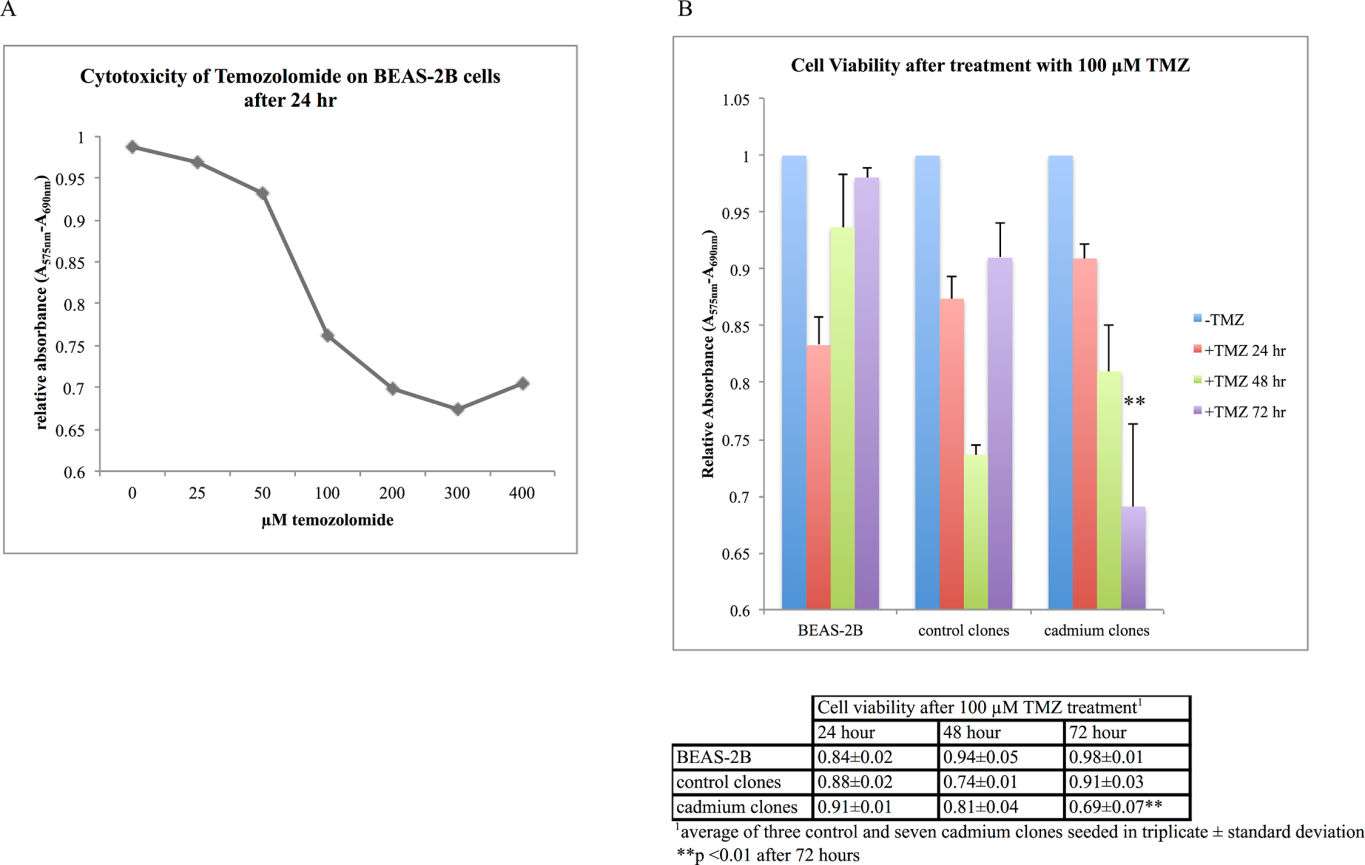
Cell viability significantly decreased in cadmium-transformed clones after 72 hour treatment with TMZ. (A) Cytotoxicity of TMZ on BEAS-2B cells after 24 hours as measured using the MTT assay. Data represents cells seeded in triplicate wells. (B) TMZ treatment decreased viability of cadmium clones relative to control clones after 72 hour treatment with TMZ. By 72 hours, BEAS-2B and control clones nearly restored to normal levels, while cadmium clone viability decreased steadily over time, indicating diminished ability to repair alkylated damage induced by TMZ. **p < 0.01 for all cadmium clones versus control clones after 72 hour TMZ treatment. Data represents three control clones and seven cadmium clones seeded in triplicate wells.

Cadmium and control clones were treated with 100 μM temozolomide (TMZ), an alkylating chemotherapeutic agent for 24, 48, or 72 hours and assessed for cell viability using the MTT assay. The cytotoxicity of normal BEAS-2B cells treated with varying doses of TMZ for 24 hours is shown in Fig 6A. 100 μM was chosen for treatment of clones as this dose has been measured in the serum of patients undergoing chemotherapy with TMZ [26]. After 72 hours, a significant reduction in cadmium clone cell viability versus control clone viability was observed (Fig 6B). The viability of BEAS-2B cells and control clones had nearly recovered to the levels of untreated cells.

## Discussion

The mechanisms of cadmium-induced carcinogenesis have yet to be fully elucidated, despite its characterization as a class I human carcinogen by IARC [5]. Cadmium is a known human lung carcinogen, and smokers are exposed to quite high doses, thus it is critical that a more thorough understanding of the mechanisms underlying carcinogenesis by cadmium be obtained. In the current study it was found that three sub-toxic doses of cadmium (0.01, 0.05, and 0.1 μM) were able to induce significant malignant transformation in normal human bronchial epithelial cells after six weeks of treatment. Previous transformation studies used much higher doses that may not be relevant to chronic human exposures. Cadmium was found to induce malignant transformation of BEAS-2B cells after 26-week treatment with 5 μM cadmium [27]. Other investigators observed a significant number of soft agar colonies after treatment with 1–2 μM cadmium for eight to twenty weeks [9,28]. In the current study, all cadmium clones exhibited increased migration ability relative to control clones and parental BEAS-2B cells, as evaluated using the scratch test. The scratch test is an *in vitro* method that allows the study of cell migration. After a gap is made in the monolayer, cells on the edge of the gap will move to reestablish cell-to-cell contacts. This mimics migration of cells *in vivo* [29]. Typically non-transformed cells migrate in a more orderly manner that is relatively slower than transformed cells. Results indicate that cadmium exposure, even at low doses, can lead to increased migration with the scratch test.

Among the top gene ontology (GO) terms associated with upregulated genes were many terms related to embryonic development or morphogenesis. Cancer cells are known to dedifferentiate and re-express embryonic genes [30]. SATB2 and noggin are two such important embryonic genes that were upregulated in cadmium-transformed clones. Noggin binds and inactivates members of the transforming growth factor beta (TGFβ) superfamily of signaling proteins [31]. IPA found TGFβ to be one of the top upstream regulators associated with genes altered in cadmium clones. TGFβ controls proliferation, differentiation, and chemotaxis and can act as a positive or negative regulator of other growth factors. In addition, many immune system-related terms were also enriched. Three toll-like receptors (TLRs) were upregulated in cadmium clones (TLR3, 4, 6). TLRs are mostly expressed by immune system cells; however, they are also expressed by epithelial cells, as these cells are exposed to microbes and involved in defense against infection. Recent studies have observed TLR expression in tumor cells, including those of the lung [32,33], thus TLR upregulation may be an important event in the transformation of lung epithelial cells. Other GO terms associated with upregulated genes included those that involve cell movement, lipid transport, nucleosome and chromatin assembly, as well as the inflammatory response.

Top GO terms associated with downregulated genes included regulation of RNA metabolism, regulation of transcription, and complement and inflammatory response activation. RNA metabolism involves any pathway or chemical reaction relating to RNA, including RNA splicing, production of small RNAs, or RNA processing. Epithelial splicing regulatory protein 2 (ESRP2), downregulated in cadmium clones, is an epithelial mRNA splicing factor necessary for normal mRNA processing that functions as a tumor suppressor [34]. Paired related homeobox 2 (PRRX2) is involved in RNA metabolism and transcription regulation and was downregulated in cadmium clones. PRRX2 plays a role in craniofacial morphogenesis and cell proliferation [35]. Hypermethylated in cancer 1 (HIC1) was also downregulated in cadmium clones. This transcriptional repressor with multiple targets is thought to be a tumor suppressor, as it is often silenced by hypermethylation in cancers [36]. Downregulation of these genes, several of which have been identified as tumor suppressors, may upregulate or otherwise alter the expression of genes that could promote transformation.

Many cancer-related networks were associated with genes altered in cadmium clones including those involved in cell growth and proliferation, as well as cellular and skeletal development and embryonic development. The top molecular functions associated with the dysregulated genes included cellular movement, small molecule biochemistry, and cell growth and proliferation. The deregulation of genes that affected cell growth, cell movement, and cell signaling, as well as the overexpression of embryonic genes all contributed to the malignant transformation of BEAS-2B cells.

SATB2, an embryonically expressed protein that regulates craniofacial development and cortical neuron differentiation, was found to be upregulated in all cadmium clones. SATB2 has various roles in transcription regulation and differentiation. SATB2 also acts as a docking site for chromatin-remodeling enzymes such as histone acetylases and deacetylases [37]. Knockout studies revealed that SATB2 enhances the activity of Runx2 and ATF4, important transcription factors that regulate osteoblast differentiation. SATB2 additionally modulates the expression of several homeobox (Hox) genes, which are essential for normal vertebrate embryonic development, including Hoxa2, an inhibitor of bone formation and regulator of bronchial arch patterning [38]. Here it was found that several Hoxb genes were significantly upregulated in transformed clones, including Hoxb2, 3, 4, 5, 6, 9, and 13. SATB2 is expressed in some intestinal epithelial cells and in a subset of neuronal cells, but is not known to be expressed in other adult tissues. Its overexpression has been reported in human cancers including those of the ovary [39], osteosarcomas [40], hepatocellular carcinomas [41] as well as in metastases [42]. SATB2 has also been found to be downregulated in certain gastric cancers where it may function as a tumor suppressor [43,44].

SATB2 mRNA was found to be upregulated in BEAS-2B clones transformed by nickel, chromium (VI), arsenic and vanadium, despite the alteration of a distinct set of genes by each metal [13]. This current study found SATB2 to be upregulated in BEAS-2B clones transformed by cadmium. SATB2 is not expressed in parental BEAS-2B cells, suggesting that its upregulation may be involved in metal carcinogenesis by modulating the expression of its downstream target genes. Overexpression of SATB2 in normal BEAS-2B cells was found to significantly increase anchorage-independent growth [25], highlighting the importance of this gene in malignant transformation. To investigate the role of SATB2 in transformation by cadmium, SATB2 was stably knocked down using shRNA. Knocking down SATB2 in a cadmium clone that highly expressed SATB2 resulted in significant inhibition of growth in soft agar. Additional studies by our lab indicated that shRNA knockdown of SATB2 in a nickel-transformed clone was also able to inhibit soft agar growth [25]. Additionally, many of the genes that were upregulated by cadmium treatment were similarly upregulated by SATB2 overexpression. The GO terms associated with these commonly upregulated genes were related to cell adhesion, growth factor signaling, and transcription regulation, which are all important pathways for carcinogenesis. These results indicate the SATB2 may be an important driver of metal-induced carcinogenesis by playing a critical role in cell survival, growth, and metastasis.

Cadmium-transformed clones exhibited increased oxidative stress markers relative to control clones including a decrease in GSH/GSSG ratios. Oxidized GSH is the major endogenous antioxidant produced by cells, involved in the neutralization of free radicals and other ROS. GSSG is generated via the formation of a disulfide bond between two molecules of GSH. Glutathione reductase recycles GSSG to GSH with simultaneous oxidation of beta nicotinamide adenine dinucleotide phosphate. When cells are under increased levels of oxidative stress, GSSG accumulates and the ratio of GSH to GSSG decreases [45]. Additionally, the levels of carbonylated protein were significantly increased in cadmium clones. ROS have the ability to induce damage to biomolecules, including proteins. Protein carbonylation is considered a hallmark of oxidative stress-induced damage [46]. An increase in autophagy marker LC3A/B-II was also observed in cadmium clones. Autophagy is a cellular survival mechanism involving lysosomal degradation of cellular components, damaged organelles, misfolded proteins, and other toxic compounds, reducing oxidative stress and protecting cells from damage. Autophagy can promote tumor growth by helping cells to adapt and survive in stressful conditions, such as under oxidative stress conditions [47,48]. ROS can induce damage to DNA, proteins, lipids and other biomolecules and oxidative stress is known to promote cancer cell growth. Thus, cadmium-transformed clones may be more able to adapt and survive under increased oxidative stress due to the increase in autophagy. shRNA knockdown of SATB2 reduced the amount of carbonylated protein present, however this decrease was not significant (p = 0.07).

MGMT levels were entirely depleted in seven of eight cadmium clones at the mRNA level, while no MGMT protein was detected in any of the transformed clones. MGMT is a critical repair enzyme that repairs the alkylated base O^6^-methylguanine (O^6^-meG). When left uprepaired, O^6^-meG mispairs with thymine instead of cytosine, causing a G:C to A:T transition mutation. MGMT covalently transfers the alkyl group of O^6^-meG to a conserved cysteine within its active site. It is considered a “suicide enzyme” as the transfer of an alkyl group to its active site renders it inactive and subject to ubiquitin-mediated degradation. MGMT can be depleted in cells as each molecule can only repair one alkylated base. Its expression can be downregulated or silenced epigenetically in cancers, typically via promoter hypermethylation [49–52]. To assess whether MGMT was epigenetically silenced in cadmium clones, clones were treated with inhibitors of DNA methylation (5-aza-2'-deoxycytidine) or histone deacetylation (sodium butyrate) and MGMT mRNA levels were measured. MGMT mRNA was observed to increase in cadmium clones and remain unchanged in control clones, indicating that MGMT was epigenetically silenced in cadmium clones. The combination of inhibitors led to increased MGMT mRNA, indicating that DNA methylation and histone acetylation may both be involved in MGMT silencing in cadmium-transformed clones.

To test the effect of diminished MGMT levels, cells were treated with TMZ for 24, 48, or 72 hours and cell viability was measured using MTT assay. TMZ is an alkylating chemotherapeutic agent used in the treatment of gliomas that undergoes spontaneous decomposition at physiological pH to 5-(3-methyl-1-triazeno)imodazole-4-carboxamide. TMZ methylates the O^6^ and N^7^ position of guanine and the N^3^ position of adenine, inducing alkylated bases, including O^6^-meG. Cells accrue O^6^-meG and other alkylated adducts, leading to continuous cycles of mismatch repair, with eventually leads to strand breaks and apoptosis [26]. Cells that overexpress MGMT are resistant to treatment with TMZ, while inhibition of MGMT in cancer cells can increase the efficacy of treatment. Treatment with 100 μM TMZ significantly reduced the viability of cadmium clones after 72 hours of treatment, indicating a diminished capacity to repair the alkylated DNA damage induced by TMZ.

## Conclusion

Low dose cadmium treatment caused significant malignant transformation in BEAS-2B cells and altered the expression of many genes in cadmium-transformed clones. SATB2 was significantly upregulated in BEAS-2B clones transformed by cadmium and shRNA knockdown of SATB2 significantly inhibited growth in soft agar. Cadmium clones exhibited increased oxidative stress and increased levels of autophagy. Additionally, the repair protein MGMT was depleted in nearly all cadmium clones and these clones were less able to repair alkylated damage induced by treatment with TMZ. Results indicate various molecular mechanisms of cadmium-induced malignant transformation in BEAS-2B cells, including upregulation of SATB2, downregulation of MGMT, and increased oxidative stress and autophagy.

This study showed that very low doses of cadmium could induce significant malignant transformation in BEAS-2B cells. RNA-seq for the first time outlined hundreds of genes altered by this metal, providing novel insight into the many genes and pathways that may be involved in carcinogenesis. SATB2, an embryonic transcription regulator found previously to be upregulated in BEAS-2B cells transformed by metal exposure was found to also be significantly upregulated after cadmium exposure. MGMT, a critical DNA repair enzyme, was nearly depleted in all transformed clones, and this depletion lead to a diminished ability to repair alkylated DNA damage. The effect of cadmium exposure on MGMT expression has not been reported in BEAS-2B cells and certainly warrants further investigation. This study provided many previously unreported potential mechanisms by which cadmium may exert its carcinogenic potential on human cells.

## Supporting Information

S1 Fig SATB2 knockdown induced a phenotypic change in cadmium-transformed clone. shRNA-1 knockdown cells appeared more cuboid and rounded then the unstransfected and scramble vector-transfected cadmium clone cells.
(TIFF)

S2 Fig MGMT levels decreased only slightly over time with chronic cadmium treatment at the protein (A) and mRNA (B) levels.
(TIFF)

S1 File Uncropped Western blots. (DOCX)

S1 Table DAVID (Database for annotation, visualization, and integrated discovery) analysis of upregulated genes in cadmium clones versus control clones. # refers to the number of genes involved.
(DOCX)

S2 Table DAVID (Database for annotation, visualization, and integrated discovery) analysis of downregulated genes in cadmium clones versus control clones. # refers to the number of genes involved.
(DOCX)

S3 Table DAVID analysis of genes commonly upregulated by SATB2 overexpression and in cadmium-transformed clones. # refers to the number of genes involved.
(DOCX)
