# Supplementary figures and images for: Molecular Mechanisms of Malignant Transformation by Low Dose Cadmium in Normal Human Bronchial Epithelial Cells
Source: PLoS One. 2016 May 17;11(5):e0155002. doi: 10.1371/journal.pone.0155002 (PMC4871351; doi:10.1371/journal.pone.0155002)

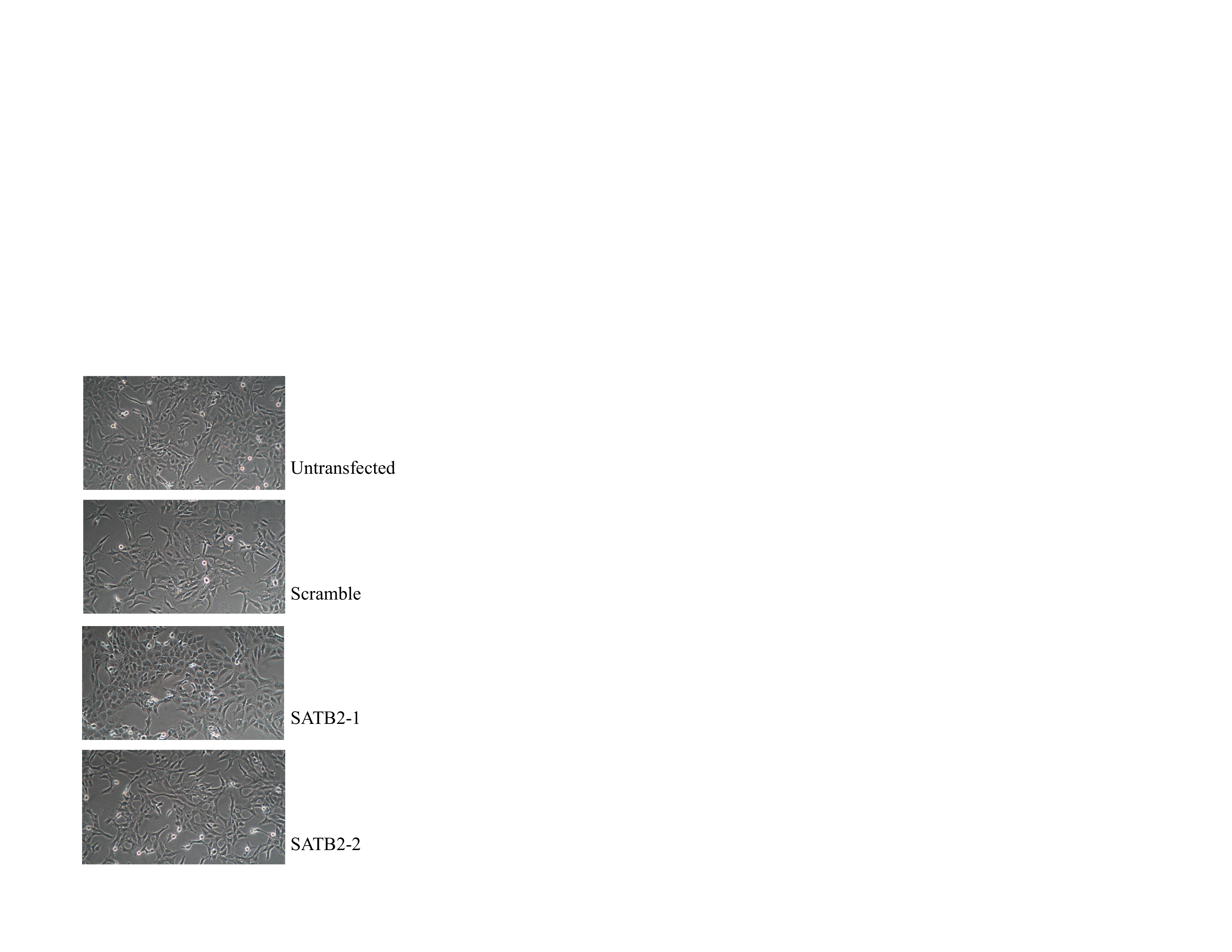

Supplement: S1 Fig — shRNA-1 knockdown cells appeared more cuboid and rounded then the unstransfected and scramble vector-transfected cadmium clone cells. (TIFF) [file pone.0155002.s001.tiff]

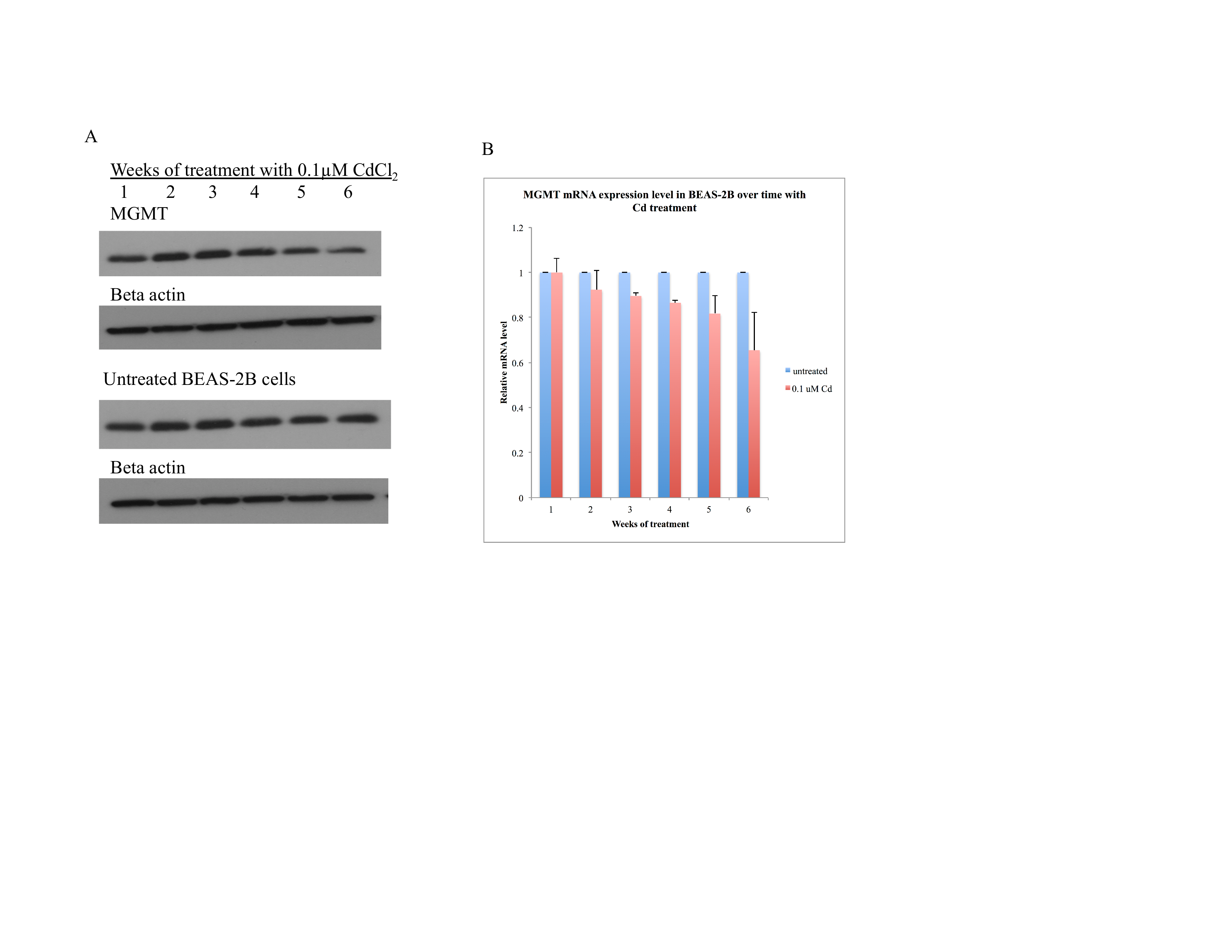

Supplement: S2 Fig — MGMT levels decreased only slightly over time with chronic cadmium treatment at the protein (A) and mRNA (B) levels. (TIFF) [file pone.0155002.s002.tiff]

Uncropped Western blots

SATB2


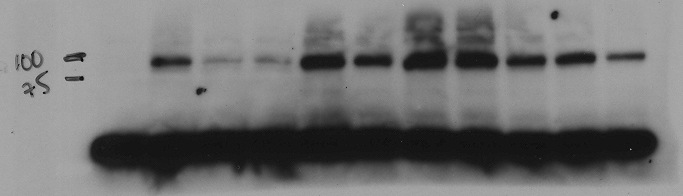


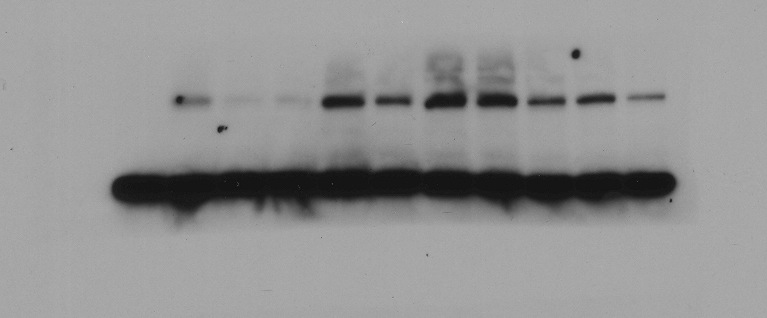


beta actin


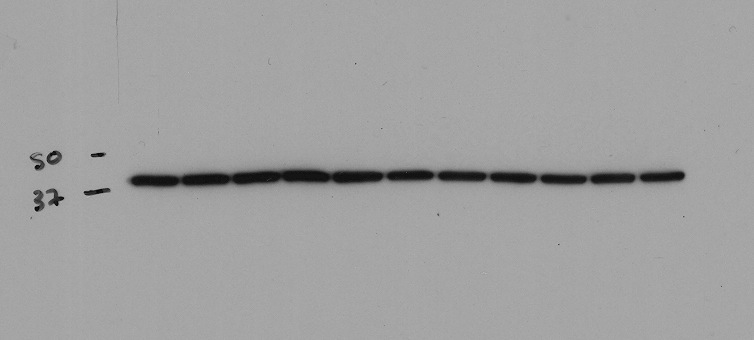


LC3A/B


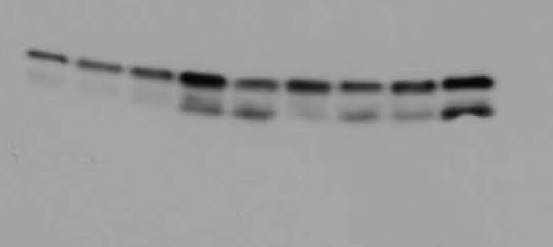


Beta actin


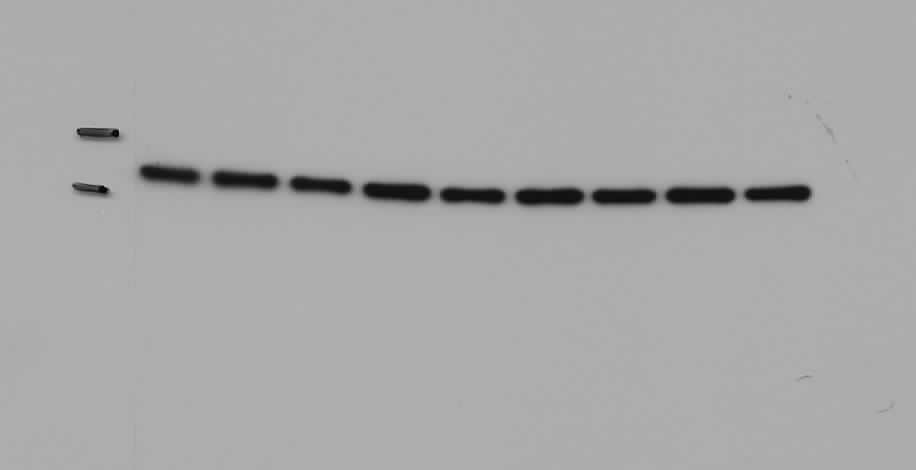


MGMT


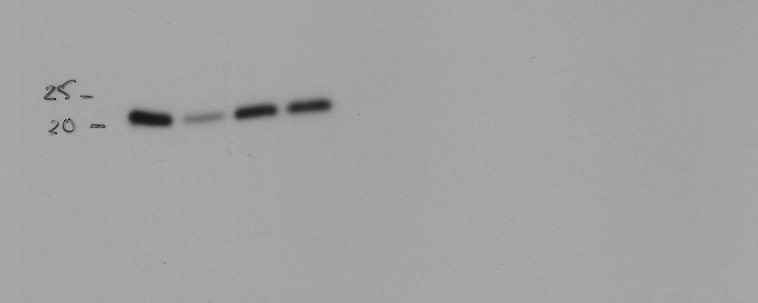


beta actin


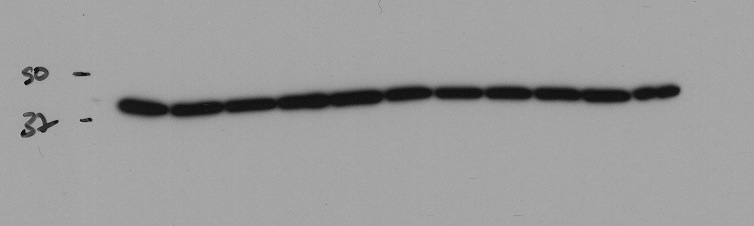


MGMT


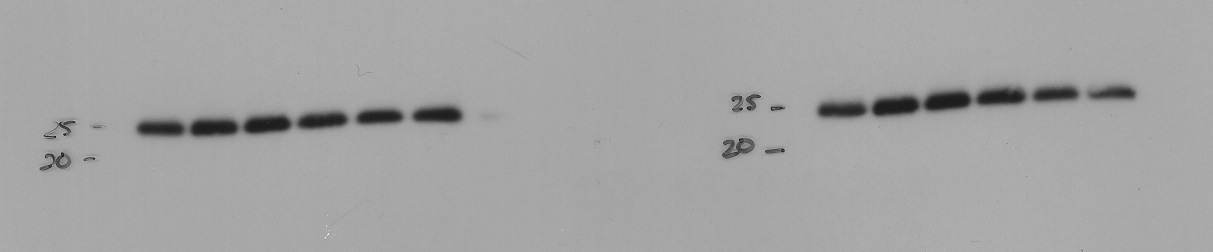


Beta actin


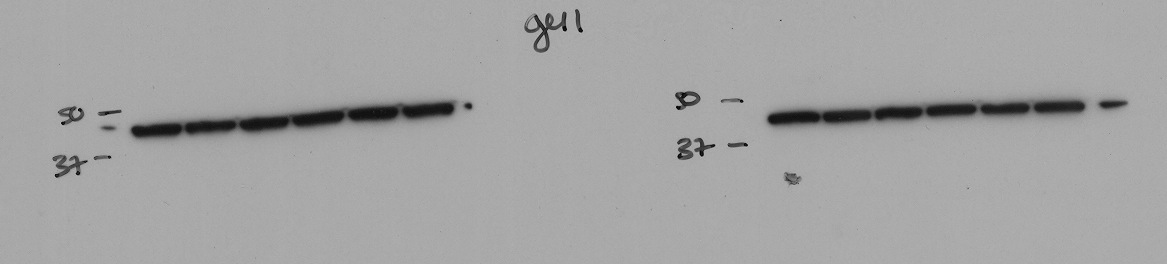

Supplement: S1 File — (DOCX) [file pone.0155002.s003.docx]
