# Supplementary material for: Molecular Mechanisms of Malignant Transformation by Low Dose Cadmium in Normal Human Bronchial Epithelial Cells
Source: PLoS One. 2016 May 17;11(5):e0155002. doi: 10.1371/journal.pone.0155002 (PMC4871351; doi:10.1371/journal.pone.0155002)
Supplement: S1 Table — # refers to the number of genes involved. (DOCX) [file pone.0155002.s004.docx]

| GO Term | # | p Value | Genes | |
| --- | --- | --- | --- | --- |
| embryonic skeletal system development | 9 | 2.15E-04 | HOXB2, 3, 4, 5, 6, 9, SATB2, NOG, KIAA1217 | |
| embryonic organ morphogenesis | 11 | 5.22E-04 | HOXB2, 3, 4, 5, 6, SATB2, NOG, ALDH1A3, FOXG1, SOBP, GFI1 | |
| skeletal system morphogenesis | 10 | 6.10E-04 | HOXB2, 3, 4, 5, 6, SATB2, P2RX7, NOG, COL13A1, PDGFRB | |
| locomotory behavior | 16 | 7.41E-04 | SNCG, PLD1, EFNB3, PDGFB, CXCL2, ATP1A3, SOBP, CXCL6, ATP7A, ROBO1, ABAT, PDGFRB, AMOT, ROBO3, RASD2, NOVA1 | |
| embryonic skeletal system morphogenesis | 7 | 0.001244 | HOXB3, HOXB4, SATB2, NOG, HOXB2, HOXB5, HOXB6 | |
| skeletal system development | 17 | 0.001245 | SATB2, NOG, FGFR3, COL13A1, PTH1R, SHOX2, HOXB2, 3, 4, 5, 6, 9, ATP7A, INHBA, HOXB4, P2RX7, PDGFRB, KIAA1217, | |
| integrin-mediated signaling pathway | 7 | 0.003578 | ADAM11, ADAM23, ITGB8, ITGA7, ADAM33, CIB2, ITGBL1 | |
| embryonic organ development | 11 | 0.003614 | HOXB2, 3, 4, 5, 6, SATB2, NOG, ALDH1A3, FOXG1, SOBP, GFI1 | |
| lipid transport | 10 | 0.003688 | SOAT2, ABCA7, P2RX7, ACE, SPNS2, STARD4, ATP9A, OSBPL7, BDKRB2, ATP8B3 | |
| positive regulation by organism of immune response of other organism during symbiotic interaction | 3 | 0.004270 | TLR3, TLR4, TLR6 | |
| positive regulation by organism of innate immunity in other organism during symbiotic interaction | 3 | 0.004270 | TLR3, TLR4, TLR6 | |
| modulation by organism of immune response of other organism during symbiotic interaction | 3 | 0.004270 | TLR3, TLR4, TLR6 | |
| positive regulation by symbiont of host immune response | 3 | 0.004270 | TLR3, TLR4, TLR6 | |
| modulation by symbiont of host immune response | 3 | 0.004270 | TLR3, TLR4, TLR6 | |
| response to wounding | 22 | 0.004279 | NOG, PDGFB, TNC, CXCL2, TLR3, HOXB13, AFAP1L2, TLR4, CXCL6, BDKRB2, TLR6, SIGIRR, HDAC5, TNFRSF1B, P2RX7, UNC13D, DYSF, THBD, AOX1, NFATC4, SERPINA1, RTN4RL2 | |
| behavior | 20 | 0.005255 | SNCG, PLD1, EFNB3, PDGFB, IL18, MAOA, CXCL2/6, ATP1A3, SOBP, KCNIP3, ATP7A, ROBO1, ABAT, PDGFRB, AMOT, ROBO3, GFI1, RASD2, NOVA1 | |
| blood vessel development and morphogenesis | 12 | 0.005304 | ATP7A, ACE, ROBO1, IL18, ITGA7, MMP19, ROBO4, AMOT, PLCD1, ANPEP, TNFSF12, TNFAIP2 | |
| lipid localization | 10 | 0.006187 | SOAT2, ABCA7, P2RX7, ACE, SPNS2, STARD4, ATP9A, OSBPL7, BDKRB2, ATP8B3 | |
| response to host immune response | 3 | 0.006316 | TLR3, TLR4, TLR6 | |
| regulation of interleukin-6 production | 5 | 0.006767 | P2RX7, TLR3, AFAP1L2, TLR4, TLR6 | |
| cell activation during immune response | 5 | 0.006767 | ATP7A, UNC13D, TLR3, TLR4, TLR6 | |
| vasculature development | 13 | 0.007219 | | IL18, MMP19, ANPEP, TNFSF12, DLX3, ATP7A, ACE, ROBO1, ITGA7, ROBO4, AMOT, PLCD1, TNFAIP2 |
| nucleosome assembly | 7 | 0.008689 | | HIST1H2AC, HIST1H2BD, HIST1H1C, HIST2H2BE, H2AFY2, HIST1H2BJ, HIST1H4H |
| inflammatory response | 15 | 0.009323 | | HDAC5, TNFRSF1B, P2RX7, UNC13D, CXCL2, AOX1, TLR3, AFAP1L2, NFATC4, TLR4, SERPINA1, CXCL6, BDKRB2, TLR6, SIGIRR |
| chromatin assembly | 7 | 0.010246 | | HIST1H2AC, HIST1H2BD, HIST1H1C, HIST2H2BE, H2AFY2, HIST1H2BJ, HIST1H4H |

**Table 1.** DAVID (Database for annotation, visualization, and integrated discovery) analysis of upregulated genes in cadmium clones versus control clones. # refers to the number of genes involved.
