# Supplementary material for: Molecular Mechanisms of Malignant Transformation by Low Dose Cadmium in Normal Human Bronchial Epithelial Cells
Source: PLoS One. 2016 May 17;11(5):e0155002. doi: 10.1371/journal.pone.0155002 (PMC4871351; doi:10.1371/journal.pone.0155002)
Supplement: S2 Table — # refers to the number of genes involved. (DOCX) [file pone.0155002.s005.docx]

| GO Term | # | p Value | Genes |
| --- | --- | --- | --- |
| Feeding behavior | 3 | 0.01152 | FOS, NPW, NMU |
| regulation of RNA metabolism | 10 | 0.02137 | KCNH1, FOS, ZNF718, ZNF783, ZNF717, KCNH7, PRRX2, ESRP2, ZNF347, HIC1 |
| regulation of transcription, DNA-dependent | 9 | 0.05003 | KCNH1, FOS, ZNF718, ZNF783, ZNF717, KCNH7, PRRX2, ZNF347, HIC1 |
| complement activation, classical pathway | 2 | 0.06644 | C3, C1R |
| complement activation | 2 | 0.09482 | C3, C1R |
| activation of plasma proteins involved in acute inflammatory response | 2 | 0.09697 | C3, C1R |

**Table 2.** DAVID (Database for annotation, visualization, and integrated discovery) analysis of downregulated genes in cadmium clones versus control clones. # refers to the number of genes involved.
