# Supplementary material for: Molecular Mechanisms of Malignant Transformation by Low Dose Cadmium in Normal Human Bronchial Epithelial Cells
Source: PLoS One. 2016 May 17;11(5):e0155002. doi: 10.1371/journal.pone.0155002 (PMC4871351; doi:10.1371/journal.pone.0155002)
Supplement: S3 Table — # refers to the number of genes involved. (DOCX) [file pone.0155002.s006.docx]

| GO Term | # | P Value | Genes |
| --- | --- | --- | --- |
| cell adhesion | 9 | 0.006227862 | PVRL4, SRPX, PCDHB5, PKP1, CD33, COL27A1, PSTPIP1, LEF1, BOC |
| biological adhesion | 9 | 0.006280463 | PVRL4, SRPX, PCDHB5, PKP1, CD33, COL27A1, PSTPIP1, LEF1, BOC |
| negative regulation of transcription from RNA polymerase II promoter | 5 | 0.021434233 | CIITA, SATB2, LEF1, PKIA, KCNIP3 |
| positive regulation of epidermal growth factor receptor signaling pathway | 2 | 0.04305858 | TGFA, AFAP1L2 |
| neurotransmitter transport | 3 | 0.04333165 | SLC1A3, SERPINB7, SLC6A17 |
| response to inorganic substance | 4 | 0.048403801 | KCNMA1, SLC1A3, LEF1, SERPINA1 |
| negative regulation of transcription, DNA-dependent | 5 | 0.053327424 | CIITA, SATB2, LEF1, PKIA, KCNIP3 |

**Table 3**. DAVID analysis of genes commonly upregulated by SATB2 overexpression and in cadmium-transformed clones. # refers to the number of genes involved.
